# Spatially Oriented S-Scheme and Schottky Junction in In_2_S_3_/Ti_3_C_2_/TiO_2_ Ternary Heterojunction for Efficient Photocatalytic H_2_ Production

**DOI:** 10.3390/molecules31101751

**Published:** 2026-05-20

**Authors:** Wenyu Liu, Defa Liu, Bin Sun, Xingpeng Liu, Pengfei Gao, Xiao Lin, Guowei Zhou

**Affiliations:** 1 Key Laboratory of Fine Chemicals in Universities of Shandong, Jinan Engineering Laboratory for Multi-Scale Functional Materials, School of Chemistry and Chemical Engineering, Qilu University of Technology (Shandong Academy of Sciences), Jinan 250353, China; wenyuliu@qlu.edu.cn (W.L.); 10431241130@stu.qlu.edu.cn (D.L.); 10431231065@stu.qlu.edu.cn (X.L.); 10431251216@stu.qlu.edu.cn (P.G.); 2 Shandong Institute of Mechanical Design and Research, School of Mechanical Engineering, Qilu University of Technology (Shandong Academy of Sciences), Jinan 250353, China; 3 College of Chemistry and Chemical Engineering, Jishou University, Jishou 416000, China; linxiao2017@whu.edu.cn

**Keywords:** ternary heterojunction, S-scheme heterojunction, Schottky junction, synergetic charge separation, photocatalytic H_2_ production

## Abstract

The reasonable structural design and interfacial modification of heterojunction photocatalysts for accelerated charge separation and boosting photocatalytic activity remains a crucial challenge in solar-driven water splitting for H_2_ production. Herein, a hierarchical structured In_2_S_3_/Ti_3_C_2_/TiO_2_ ternary heterojunction was effectively constructed through a facile hydrothermal method integrated with a self-assembly strategy, in which Ti_3_C_2_ and TiO_2_ were loaded on the surface of hierarchical In_2_S_3_ microspheres assembled from nanosheets. In the photocatalytic system, the in situ electron paramagnetic resonance verifies that the photogenerated charge transfer between In_2_S_3_ and TiO_2_ obeys a typical S-scheme mechanism. Meanwhile, the introduction of Ti_3_C_2_ MXene as a conductive cocatalyst further promotes the separation and transfer of photogenerated charge through the formation of a Schottky junction, thus remarkably boosting the photocatalytic performance. Under simulated sunlight irradiation, the In_2_S_3_/Ti_3_C_2_/TiO_2_ ternary heterojunction exhibits a superior H_2_ production rate compared to pure TiO_2_ and In_2_S_3_. Moreover, the ternary heterojunction also displays outstanding stability after five consecutive cycling tests. This work highlights the synergistic integration of an S-scheme and Schottky junction in a ternary heterostructure for efficient charge separation, providing a feasible strategy for designing high-performance photocatalysts toward solar-driven H_2_ production.

## 1. Introduction

With the development of society, the energy shortage caused by the rapid consumption of fossil fuels has become an unavoidable issue [1,2]. In order to solve the issue, researchers have developed some alternative energy sources [3]. Hydrogen energy is expected to become a major energy source in the future due to its high energy density, green environmental protection, and high efficiency [4,5]. Converting solar energy into hydrogen energy by water splitting is one of the most sustainable solutions for producing H_2_, helping to meet the ever-increasing global energy demand [6,7]. Nowadays, metal sulfides have been widely employed in photocatalysis owing to their visible-light response, facile synthesis, unique photoelectric properties, and low cost [8,9]. Among them, In_2_S_3_ stands out as a promising photocatalytic candidate, featuring a broad light absorption range, low cost, excellent carrier mobility, and a suitable band gap [10,11]. Nevertheless, the rapid recombination of photogenerated charge, low quantum efficiency, and inevitable photocorrosion significantly impede photocatalytic activity. Thus, designing a high-performance photocatalyst is crucial for promoting the development of photocatalytic technology.

To solve these bottlenecks, extensive efforts have been devoted to modifying photocatalysts, including doping, loading cocatalysts, vacancy engineering, heterojunction construction, etc. [12,13,14,15]. Particularly, the construction of S-scheme heterojunctions has received widespread interest [16,17]. Based on the S-scheme heterojunction formation mechanism, it has been found that In_2_S_3_ possesses a suitable energy band structure and can act as a reduction photocatalyst to couple with TiO_2_ for the construction of an S-scheme heterojunction, which contributes to accelerating the separation of photogenerated charge and significantly enhances photocatalytic activity. Furthermore, the 2D TiO_2_ nanomaterial has excellent photocatalytic activity [18,19]. Therefore, the construction of an S-scheme heterojunction with a 2D structure can also effectively shorten the charge migration distance, provide a larger specific surface area, and expose abundant active sites [20,21].

To further boost photocatalytic performance, the loading cocatalysts on the photocatalyst surface have also been extensively investigated [22,23]. As novel 2D materials, MXenes have been demonstrated as efficient cocatalysts in the photocatalytic field because of their excellent metallic conductivity, high hydrophilicity, and high charge mobility [24,25,26]. In particular, the surface functional groups of Ti_3_C_2_ offer abundant active sites for interfacial coupling with photocatalysts, thereby facilitating the construction of Schottky junctions with intimate interfacial contact [27,28]. Currently, researchers are coupling MXene with photocatalysts to overcome the problems of the low photogenerated charge separation efficiency and narrow light absorption range of single photocatalysts [29,30]. For example, Li et al. fabricated a ZnIn_2_S_4_/Ti_3_C_2_ composite aiming toward enhancing the photocatalytic performance. In this composite system, Ti_3_C_2_ and ZnIn_2_S_4_ were in close contact to form a Schottky junction, which greatly facilitated the separation of the photogenerated charge of ZnIn_2_S_4_, thereby improving the photocatalytic activity [31]. Liu et al. also fabricated a 2D/2D Ti_3_C_2_ MXene/CdIn_2_S_4_ composite with abundant sulfur vacancies via a solvothermal approach. The composite displayed outstanding photocatalytic activity, which can be ascribed to the formation of a Schottky junction and sulfur vacancies [32]. Therefore, constructing a hierarchical structured In_2_S_3_/Ti_3_C_2_/TiO_2_ ternary heterojunction with an S-scheme and Schottky junction is a promising strategy to overcome the inherent drawbacks of single-component and binary heterojunction photocatalysts, further boosting the photocatalytic activity of water splitting for H_2_ production. The ternary heterostructure establishes dual spatially oriented charge transfer channels, which not only retain the strong redox ability of the photogenerated carrier via the S-scheme mechanism, but also further accelerate interfacial photogenerated carrier separation through the Schottky junction induced by metallic Ti_3_C_2_ MXene. Furthermore, the hierarchical structure assembled from nanosheets provides a shortened charge migration pathway, enlarged specific surface area, and sufficient active sites. To the best of our knowledge, the synergistic design combining an S-scheme and Schottky junction in a In_2_S_3_/Ti_3_C_2_/TiO_2_ ternary heterojunction for enhanced H_2_ production has been rarely reported previously.

In the present study, we rationally fabricated a hierarchical structured In_2_S_3_/Ti_3_C_2_/TiO_2_ ternary heterojunction with an S-scheme and Schottky junction. With the introduction of Ti_3_C_2_ and TiO_2_, the ternary heterojunction exhibits remarkable photocatalytic activity, achieving a H_2_ production rate of 446.65 μmol g^−1^ h^−1^, approximately 20 and 22 times that obtained on pure In_2_S_3_ and TiO_2_, respectively. The significant enhancement in photocatalytic H_2_ production activity can be attributed to the dual heterojunction structure of the S-scheme and Schottky junction, which constructs a dual photogenerated charge transfer channel to effectively promote the separation and transfer of photogenerated electron–hole pairs, ultimately boosting the overall photocatalytic H_2_ production efficiency.

## 2. Results and Discussion

### 2.1. Morphology and Structure Analysis

**Figure 1:**
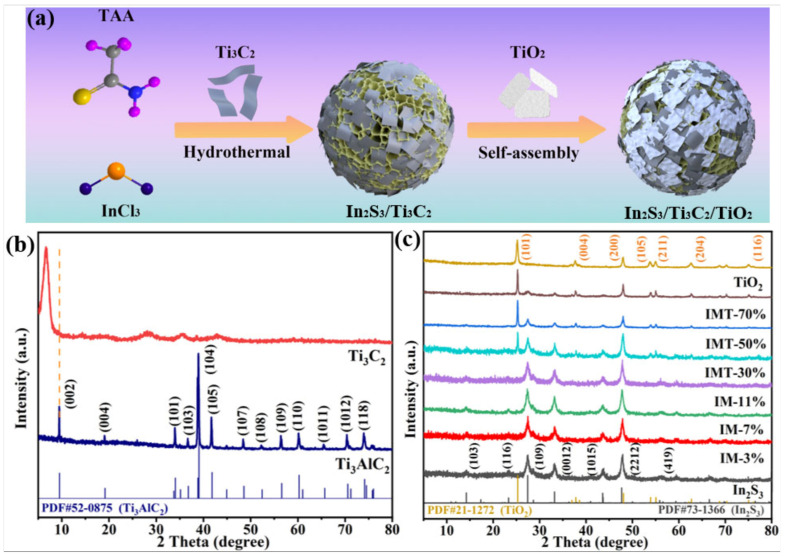
(**a**) Synthesis diagram of the hierarchical structured In_2_S_3_/Ti_3_C_2_/TiO_2_ ternary heterojunction; XRD spectra of (**b**) Ti_3_AlC_2_, Ti_3_C_2_, and (**c**) In_2_S_3_, TiO_2_, In_2_S_3_/Ti_3_C_2_, and In_2_S_3_/Ti_3_C_2_/TiO_2_.

The hierarchical structured In_2_S_3_/Ti_3_C_2_/TiO_2_ ternary heterojunction was designed and synthesized via a hydrothermal route combined with a self-assembly strategy, as shown in Figure 1a. Firstly, the Al layers in Ti_3_AlC_2_ can be etched by LiF and HCl to form few-layer Ti_3_C_2_ nanosheets [22,23,33]. Then, the In_2_S_3_/Ti_3_C_2_ heterojunction was prepared by the hydrothermal method using InCl_3_ 4H_2_O as the indium source and thioacetamide (TAA) as the sulfur source in the presence of Ti_3_C_2_ nanosheets. Furthermore, TiO_2_ nanosheets can be prepared via the solvothermal method with HF serving as a structure-regulating agent. Finally, the as-obtained In_2_S_3_/Ti_3_C_2_ and TiO_2_ nanosheets can be coupled through a self-assembly strategy, thus forming a hierarchical structured In_2_S_3_/Ti_3_C_2_/TiO_2_ ternary heterojunction.

The physical phase and crystal structure of the as-prepared samples were determined using an X-ray powder diffractometer (XRD). From Figure 1b, the diffraction peaks at 9.4°, 19.0°, 33.9°, 36.6°, 39.0°, 41.7°, 48.4°, 52.3°, 56.4°, 60.1°, 65.4°, 70.3°, and 73.9° correspond to the (002), (004), (101), (103), (104), (105), (107), (108), (109), (110), (1011), (1012), and (118) crystal planes of Ti_3_AlC_2_, respectively [22]. After the selective etching of Al layers in Ti_3_AlC_2_, the most intense diffraction peaks of Ti_3_C_2_ disappear, especially the (104) peak located at 39°. This result proves that the Al layers of Ti_3_AlC_2_ are etched. Meanwhile, the (002) peak of Ti_3_C_2_ shows broadening and a shift to a lower angle, indicating that the Ti_3_AlC_2_ has transformed into the 2D layered structure of Ti_3_C_2_ [22,23]. Figure 1c shows that the diffraction peaks at 14.2°, 23.3°, 27.5°, 33.2°, 43.7°, 47.9°, and 56.1° can been indexed in the (103), (116), (109), (0012), (1015), (2212), and (419) crystal planes of In_2_S_3_ (JCPDS NO. 25-0390), respectively [10,11]. For pure TiO_2_, the diffraction peaks at 25.2°, 37.6°, 47.9°, 53.8°, 55.0°, 62.7°, and 74.9° belong to the (101), (004), (200), (105), (211), (204), and (204) crystal planes of anatase TiO_2_ (JCPDS No. 21-1272), respectively [34]. With the introduction of Ti_3_C_2_, the diffraction peaks of In_2_S_3_ are clearly present in the In_2_S_3_/Ti_3_C_2_ heterojunction. Nevertheless, no distinct diffraction peak of Ti_3_C_2_ is detected, which is caused by the relatively homogeneous dispersion of Ti_3_C_2_ and the weak diffraction peaks of the few-layer Ti_3_C_2_ being shielded by the diffraction peaks of In_2_S_3_ [22,23]. Notably, the diffraction peaks of In_2_S_3_ and TiO_2_ are simultaneously detected in the In_2_S_3_/Ti_3_C_2_/TiO_2_ ternary heterojunction. Furthermore, the diffraction peaks of TiO_2_ are gradually enhanced as the TiO_2_ content increases, while the diffraction peaks of In_2_S_3_ are gradually weakened. These results confirm that the ternary heterojunction has been successfully constructed.

**Figure 2:**
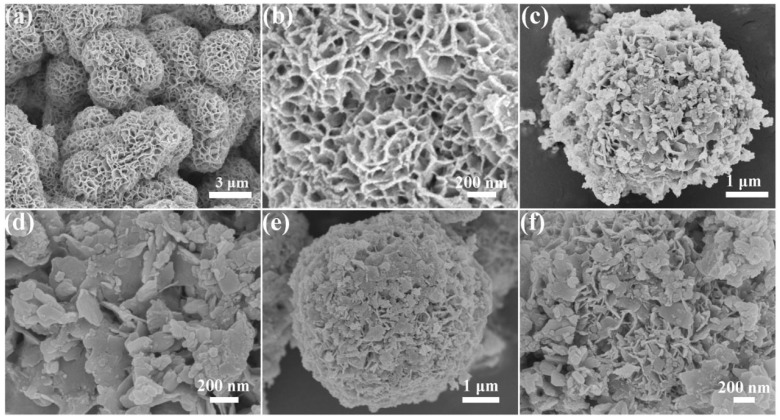
FESEM images of (**a**,**b**) In_2_S_3_, (**c**,**d**) IM-7%, and (**e**,**f**) IMT-50%.

The morphology of the as-obtained samples was acquired by field emission scanning electron microscopy (FESEM). From Figure 2a,b, hierarchical In_2_S_3_ microspheres can be clearly observed with the self-assembly of nanosheets. Pure TiO_2_ shows irregular 2D nanosheets with sizes ranging from 100 to 200 nm (Figure S1a). As shown in Figure S1b, Ti_3_C_2_ possesses a 2D layered structure composed of few-layer nanosheets, suggesting that Al layers have been successfully etched. With the introduction of Ti_3_C_2_ in the In_2_S_3_/Ti_3_C_2_ heterojunction (IM-7%), it can be seen that the Ti_3_C_2_ is successfully anchored on the surface of In_2_S_3_ (Figure 2c,d). By coupling TiO_2_ nanosheets with the In_2_S_3_/Ti_3_C_2_ heterojunction via the self-assembly strategy (IMT-50%), the surface of the In_2_S_3_/Ti_3_C_2_/TiO_2_ ternary heterojunction obviously become much denser compared to the In_2_S_3_/Ti_3_C_2_ heterojunction (Figure 2e,f). Simultaneously, the In_2_S_3_/Ti_3_C_2_/TiO_2_ ternary heterojunction still maintains a hierarchical structure similar to the In_2_S_3_ and In_2_S_3_/Ti_3_C_2_ heterojunction. The results indicate that the In_2_S_3_/Ti_3_C_2_/TiO_2_ ternary heterojunction is successfully prepared.

**Figure 3:**
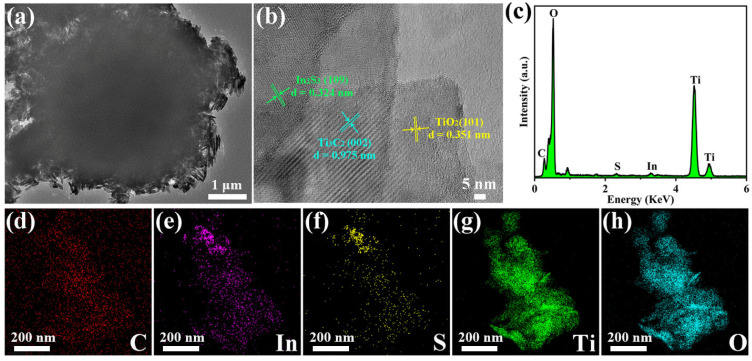
(**a**) TEM image, (**b**) HRTEM image, (**c**) EDS, and (**d**–**h**) elemental mappings of C, In, S, Ti, and O of IMT-50%.

In order to further characterize the microstructure and elemental distribution of the as-prepared samples, TEM was performed, as exhibited in Figure 3. From Figure 3a, a tightly packed microsphere can be observed in the In_2_S_3_/Ti_3_C_2_/TiO_2_ ternary heterojunction. Additionally, the high-resolution TEM (HRTEM) in Figure 3b displays clear lattice spacing at 0.351, 0.975, and 0.324 nm, which correspond to the (101), (002), and (109) crystal planes of TiO_2_, Ti_3_C_2_, and In_2_S_3_, respectively [11,22,34], indicating the presence of In_2_S_3_, TiO_2_, and Ti_3_C_2_ in the ternary heterojunction. Meanwhile, the result also suggests that a close interfacial contact is formed among In_2_S_3_, TiO_2_, and Ti_3_C_2_. Furthermore, energy dispersive spectrometry (EDS) and elemental mapping were carried out to further investigate the elemental composition and distribution. From Figure 3c–h, the coexistence and uniform distribution of C, In, O, S, and Ti can be clearly observed in the In_2_S_3_/Ti_3_C_2_/TiO_2_ ternary heterojunction, which further confirm the successful preparation of the ternary heterojunction.

**Figure 4:**
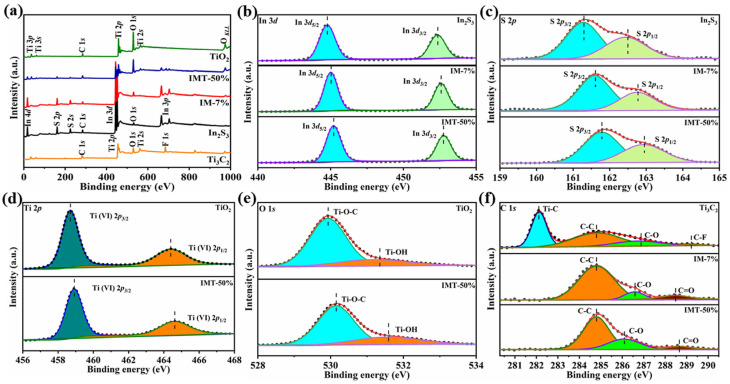
(**a**) XPS survey spectra and high-resolution XPS spectra of (**b**) In 3*d*, (**c**) S 2*p*, (**d**) Ti 2*p*, (**e**) O 1*s*, and (**f**) C 1*s* of Ti_3_C_2_, In_2_S_3_, TiO_2_, IM-7%, and IMT-50%.

The chemical composition, elemental valence states, and interfacial interactions of the as-prepared samples were characterized by X-ray photoelectron spectroscopy (XPS). From Figure 4a, IMT-50% shows the presence of In, S, Ti, C, and O compared to Ti_3_C_2_, In_2_S_3_, TiO_2_, and IM-7%, consisting with EDS and elemental mappings (Figure 3c–h). Pure In_2_S_3_ shows the two characteristic peaks at 444.8 and 452.4 eV, attributed to the In 3*d*_5/2_ and In 3*d*_3/2_, respectively (Figure 4b). Furthermore, the two characteristic peaks at 161.3 and 162.5 eV belong to S 2*p*_3/2_ and S 2*p*_1/2_, respectively (Figure 4c) [11]. After modification with the Ti_3_C_2_ nanosheet, the characteristic peaks of In 3*d* and S 2*p* in the In_2_S_3_/Ti_3_C_2_ heterojunction are positively shifted. The upward shift in binding energy signifies interfacial electron transfer among atoms and a reduced electron cloud density around the metal ions, thereby strengthening the Coulombic attraction between the indium nucleus and the emitted electrons [35]. For the In_2_S_3_/Ti_3_C_2_/TiO_2_ ternary heterojunction, the binding energies of In 3*d* and S 2*p* are also positively shifted. This result suggests a reduced surface electron density of In_2_S_3_ in the In_2_S_3_/Ti_3_C_2_/TiO_2_, indicating a heterojunction formation between In_2_S_3_ and TiO_2_, which effectively facilitates the transfer of photogenerated electrons [36]. The Ti 2*p* spectrum of TiO_2_ in Figure 4d fits two characteristic peaks at 458.7 and 464.4 eV, corresponding to Ti (VI)2*p*_3/2_ and Ti (VI)2*p*_1/2_, respectively [22,23]. It is noteworthy that the characteristic peak of Ti 2*p* for IMT-50% shifts towards a higher binding energy compared to TiO_2_, indicating that TiO_2_ is losing electrons in the heterojunction, which suggests that more electrons are transferred to Ti_3_C_2_ [22]. Similarly, Figure 4e shows that the characteristic peaks of O 1*s* over IMT-50% are shifted towards higher binding energies compared to pristine TiO_2_, which is in agreement with the Ti 2*p*. For Figure 4f, the high-resolution C 1*s* spectrum of Ti_3_C_2_ is fitted with four characteristic peaks at 282.1, 284.8, 286.9, and 289.2 eV, corresponding to Ti-C, C-C, C-O, and C-F bonds, respectively [22]. Notably, the C-O bonds of IM-7% and IMT-50% are negatively shifted, indicating an elevated electron density on the Ti_3_C_2_ surface. The variation in binding energy is ascribed to the equilibrium state of charge redistribution achieved after heterojunction formation, where an increase in binding energy implies a reduction in electron density. Therefore, it can be inferred that the formation of a built-in electric field reduces the Coulomb repulsion and enhances the photogenerated charge transfer across the interface.

### 2.2. Photoelectrochemical Performance Analysis

**Figure 5:**
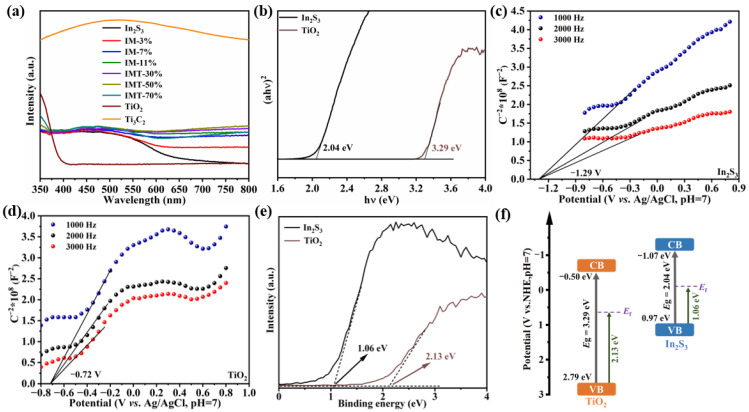
(**a**) UV–vis diffuse reflectance spectra of the as-obtained samples. (**b**) Tauc plots, (**c**,**d**) Mott–Schottky curves, (**e**) VB-XPS spectra, and (**f**) the band structure diagram of In_2_S_3_ and TiO_2_.

The light absorption performances of samples were explored via UV–vis diffuse reflectance spectra and are demonstrated in Figure 5a. Pure In_2_S_3_ demonstrates a visible light absorption ability. Compared with In_2_S_3_, the light absorption abilities of In_2_S_3_/Ti_3_C_2_ heterojunctions are greatly enhanced via the incorporation of Ti_3_C_2_ [22]. As the Ti_3_C_2_ content increases, the light absorption abilities of In_2_S_3_/Ti_3_C_2_ heterojunctions are gradually enhanced. As expected, the introduction of TiO_2_ in the In_2_S_3_/Ti_3_C_2_/TiO_2_ ternary heterojunction can further improve the light absorption ability, implying the formation of a strong interaction between In_2_S_3_, TiO_2_, and Ti_3_C_2_. By the Kubelka–Munk function, the band gaps of In_2_S_3_ and TiO_2_ are calculated to be 2.04 and 3.29 eV, respectively (Figure 5b). Furthermore, the energy band structures of In_2_S_3_ and TiO_2_ are analyzed using Mott–Schottky tests (Figure 5c,d). It is observed that the tangent slopes of In_2_S_3_ and TiO_2_ are positive, indicating that In_2_S_3_ and TiO_2_ belong to n-type semiconductors. The flat-band potentials of In_2_S_3_ and TiO_2_ are about −1.29 and −0.72 V vs. Ag/AgCl, respectively. For n-type semiconductors, the flat-band potential coincides with the CB potential [23]. Based on the formula for converting the standard hydrogen electrode potential (NHE) to the Ag/AgCl electrode potential [34], the CB potentials of In_2_S_3_ and TiO_2_ are determined as −1.07 and −0.50 eV. Meanwhile, the valence band (VB) potentials of In_2_S_3_ and TiO_2_ are also calculated to be 0.97 and 2.79 eV via the equation (*E*_g_ = *E*_VB_ − *E*_CB_) [34]. Additionally, the VB-XPS spectra was employed to identify the energy difference between *E*_VB_ and the Fermi level (*E*_f_) [37]. As shown in Figure 5e, the energy differences of In_2_S_3_ and TiO_2_ are 1.06 and 2.13 eV. With the *E*_VB_ of In_2_S_3_ and TiO_2_ determined as 0.97 and 2.79 eV, the *E*_f_ values of In_2_S_3_ and TiO_2_ correspond to −0.09 and 0.66 eV, respectively. Combined with the above analytical results of the UV–vis diffuse reflectance spectra, Mott–Schottky tests, and VB-XPS spectra, the band structure diagram of In_2_S_3_ and TiO_2_ is illustrated in Figure 5f.

**Figure 6:**
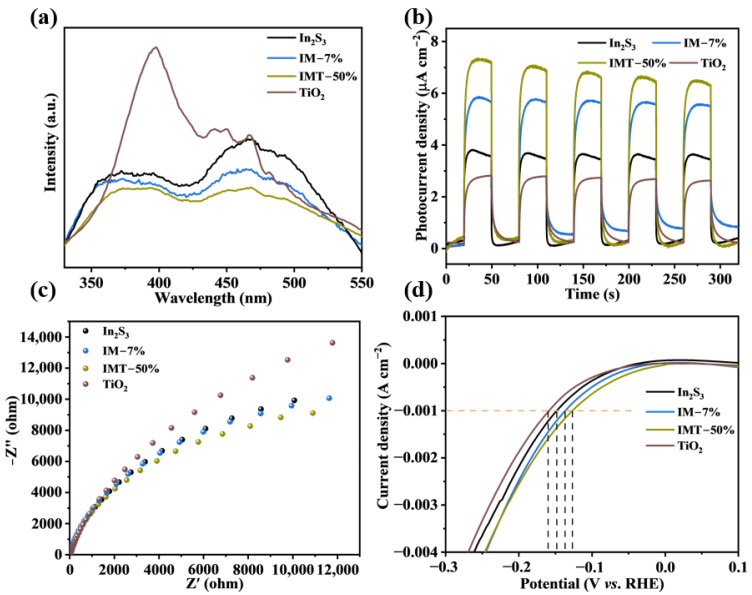
(**a**) PL spectra, (**b**) transient photocurrent response, (**c**) EIS, and (**d**) LSV tests of In_2_S_3_, TiO_2_, IM-7%, and IMT-50%.

The photogenerated charge separation and transfer efficiencies of the as-prepared samples were assessed through the photoluminescence (PL) spectra. In general, a higher intensity of PL spectra shows a higher recombination efficiency of the photogenerated charge, thus leading to lower photocatalytic activity [34]. As presented in Figure 6a, pure In_2_S_3_ and TiO_2_ show a higher PL intensity, implying the rapid recombination of photogenerated charge. With the introduction of Ti_3_C_2_ in the In_2_S_3_/Ti_3_C_2_ heterojunction, the PL intensity is significantly lower than that of pure In_2_S_3_, thus hindering the photogenerated electron–hole pairs’ recombination [36]. Interestingly, the PL intensity of the In_2_S_3_/Ti_3_C_2_/TiO_2_ ternary heterojunction can be further reduced compared to In_2_S_3_, TiO_2_, and the In_2_S_3_/Ti_3_C_2_ heterojunction. These results show that the In_2_S_3_/Ti_3_C_2_/TiO_2_ ternary heterojunction exhibits the highest separation efficiency of photogenerated charge, which endows it with outstanding photocatalytic performance. Figure 6b shows the transient photocurrent responses of In_2_S_3_, TiO_2_, IM-7%, and IMT-50%. The photocurrent intensity of IM-7% is significantly higher than that of pure In_2_S_3_, while IMT-50% exhibits an even higher photocurrent density, indicating that the introduction of Ti_3_C_2_ and TiO_2_ can enhance the charge separation efficiency in the In_2_S_3_/Ti_3_C_2_/TiO_2_ ternary heterojunction. Simultaneously, IMT-50% also exhibits a smaller EIS semicircle radius compared to pure In_2_S_3_, TiO_2_, and IM-7%, suggesting lower interfacial resistance and more efficient charge transfer across the interface (Figure 6c). Thus, the formation of the ternary heterojunction can promote more photogenerated charge to participate in the photocatalytic reaction.

Generally, the photocatalytic H_2_ production activity is highly dependent on the overpotential of the photocatalyst. An overpotential close to zero is more favorable for driving photocatalytic H_2_ production [34]. From linear sweep voltammetry (LSV) tests (Figure 6d), it is evident that IMT-50% shows the lowest overpotential, which suggests that the In_2_S_3_/Ti_3_C_2_/TiO_2_ ternary heterojunction possesses a stronger reduction capability and is more favorable for the photocatalytic H_2_ production reaction.

### 2.3. Photocatalytic H_2_ Production Performance Analysis

**Figure 7:**
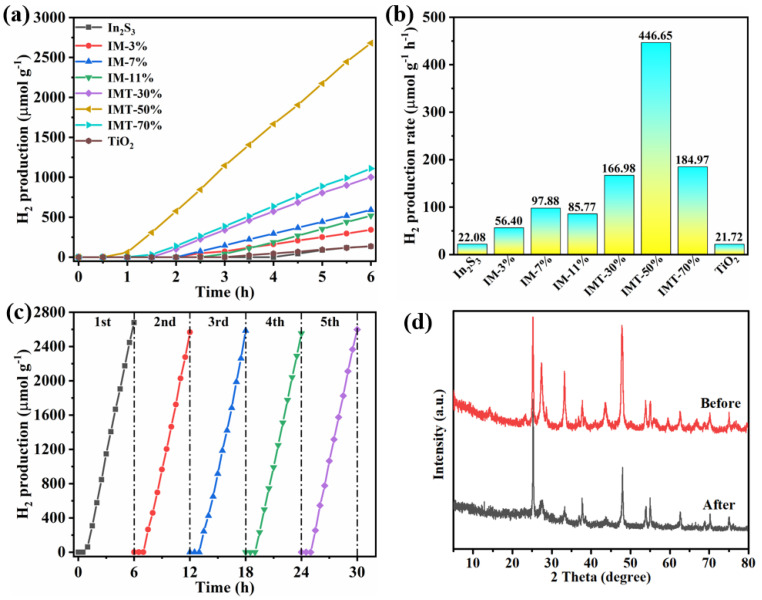
(**a**) Photocatalytic H_2_ production curves and (**b**) photocatalytic H_2_ production rates of the as-prepared samples under simulated sunlight irradiation; (**c**) recyclability experiments of IMT-50% and (**d**) XRD patterns of IMT-50% before and after photocatalytic H_2_ production experiments.

The photocatalytic H_2_ production activities of the samples were assessed under simulated sunlight irradiation. From Figure 7a,b, the lower photocatalytic H_2_ production rates of 21.72 and 22.08 μmol g^−1^ h^−1^ are observed over pure TiO_2_ and In_2_S_3_, respectively, which is due to the fast photogenerated charge recombination of the single photocatalyst [38]. When Ti_3_C_2_ is introduced into In_2_S_3_ to form the In_2_S_3_/Ti_3_C_2_ heterojunction, the photocatalytic H_2_ production activity is obviously improved. Particularly, IM-7% demonstrates a photocatalytic H_2_ production rate of 97.88 μmol g^−1^ h^−1^. This is mainly due to the role of Ti_3_C_2_ as an electron acceptor to form the Schottky junction between In_2_S_3_ and Ti_3_C_2_, which effectively traps electrons and improves the separation and transfer of photogenerated charge [27,28]. After TiO_2_ is introduced into the In_2_S_3_/Ti_3_C_2_ heterojunction, the photocatalytic H_2_ production activity of the In_2_S_3_/Ti_3_C_2_/TiO_2_ ternary heterojunction is further enhanced, reaching up to 446.65 μmol g^−1^ h^−1^, which is 20 and 22 times that of pure In_2_S_3_ and TiO_2_. This result is also significantly superior to those of other reported photocatalysts, as shown in Table S1. Owing to the broad light absorption feature of the In_2_S_3_/Ti_3_C_2_/TiO_2_ ternary heterojunction (Figure 5a), the photocatalytic H_2_ production activities of the ternary heterojunctions have also been conducted under visible light irradiation (λ > 420 nm) and are presented in Figure S2. It can be clearly observed that IMT-50% also exhibits an excellent photocatalytic H_2_ production activity of 383.22 μmol g^−1^ h^−1^. The improvement of photocatalytic activity of the In_2_S_3_/Ti_3_C_2_/TiO_2_ ternary heterojunction is attributed to the introduction of TiO_2_ to further promote the separation of photogenerated charge, thus achieving efficient photocatalytic H_2_ production activity. Additionally, the photocatalytic stability and reusability of the In_2_S_3_/Ti_3_C_2_/TiO_2_ ternary heterojunction (IMT-50%) are evaluated through recycling experiments. As shown in Figure 7c, the photocatalytic performance shows no significant decrease after five cycles. Meanwhile, no obvious structural and morphological alterations are detected via the XRD pattern and FESEM image (Figure 7d and Figure S3). The results confirm the photocatalytic stability and reusability of the In_2_S_3_/Ti_3_C_2_/TiO_2_ ternary heterojunction.

### 2.4. Mechanism of Photocatalytic H_2_ Production Activity Enhancement

**Figure 8:**
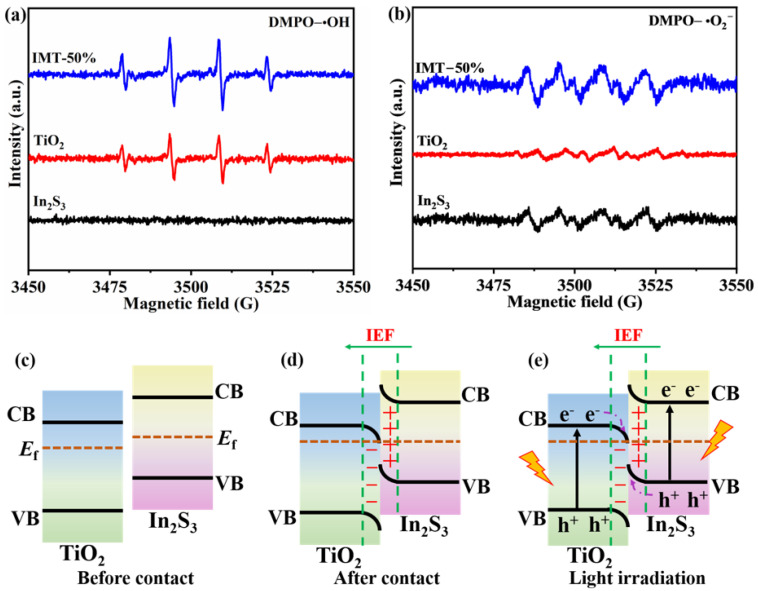
In situ EPR spectra of (**a**) DMPO-•OH and (**b**) DMPO-•O_2_^−^ of TiO_2_, In_2_S_3_, and IMT-50% under light illumination; (**c**–**e**) schematic diagram of the S-scheme mechanism of the In_2_S_3_/TiO_2_ heterojunction.

To further explore the charge transfer mechanism, the in situ electron paramagnetic resonance (EPR) spectra were detected using 5,5-dimethyl-1-pyrrolin-n-oxide (DMPO) as the spin trapping reagent [34,39]. From Figure 8a,b, the DMPO-•OH and DMPO-•O_2_^−^ signals in pure TiO_2_ are detectable, while pure In_2_S_3_ only exhibits DMPO-•O_2_^−^ signals without DMPO-•OH signals. This may be due to the fact that the VB potential (2.79 eV) and CB potential (−0.50 eV) of TiO_2_ can simultaneously meet the potential requirements of H_2_O/•OH (2.37 eV) and O_2_/•O_2_^−^ (−0.33 eV) [34,40]. The VB potential of In_2_S_3_ (0.97 eV) is lower compared to the H_2_O/•OH potential [41]. In comparison to pure TiO_2_ and In_2_S_3_, the IMT-50% displays remarkably stronger intensities of DMPO-•OH and DMPO-•O_2_^−^ signals. If the IMT-50% exhibits a type-II charge transfer behavior, the DMPO-•OH and DMPO-•O_2_^–^ signals in IMT-50% would be weaker because of its lower oxidation and reduction potentials [34], as shown in Figure S4. However, this phenomenon is contrary to the type-II heterojunction. Therefore, the charge transfer path of the In_2_S_3_/TiO_2_ heterojunction follows the S-scheme mechanism rather than the type-II heterojunction.

**Figure 9:**
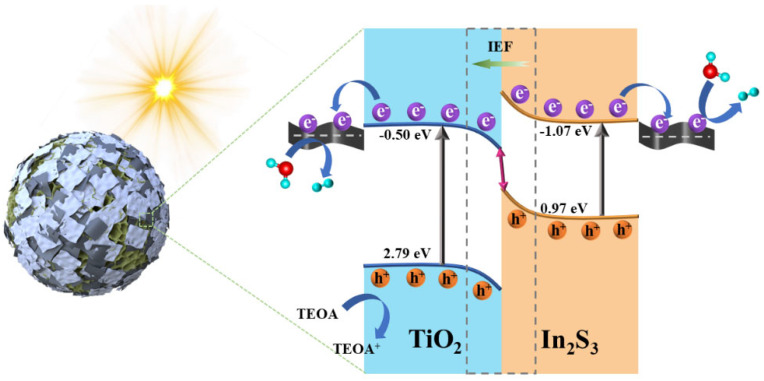
The band structure and possible photocatalytic H_2_ production mechanism of the In_2_S_3_/TiO_2_/Ti_3_C_2_ ternary heterojunction under simulated sunlight irradiation.

Based on the above analysis, the possible charge transfer mechanism of the In_2_S_3_/TiO_2_ heterojunction is proposed. As shown in Figure 8c–e, the electron in In_2_S_3_ will migrate to TiO_2_ across the contact interface due to the different *E*_f_ of In_2_S_3_ and TiO_2_, achieving a balanced *E*_f_ and forming an internal electric field (IEF) in the direction of In_2_S_3_ to TiO_2_ [42,43]. As a result, the downward and upward energy band bending occur in TiO_2_ and In_2_S_3_, respectively. Under light irradiation, the photogenerated electrons of In_2_S_3_ and TiO_2_ are stimulated to the CB, while the holes remain in the VB. By the combined influence of the IEF and energy band bending, the electrons in the CB of TiO_2_ can transfer and combine with the holes in the VB of In_2_S_3_. The results imply that the CB of In_2_S_3_ holds electrons with high reducing capacity and the VB of TiO_2_ retains strong oxidizing holes. Furthermore, owing to the metallic character of Ti_3_C_2_, it can form a Schottky junction with In_2_S_3_ and TiO_2_, and thus electrons in the In_2_S_3_ and TiO_2_ can be transferred to the Ti_3_C_2_ (Figure 9). The photogenerated electrons gathered on the Ti_3_C_2_ reduce H_2_O into H_2_, while the photogenerated holes in the TiO_2_ can oxidize triethanolamine (TEOA) into by-products [44,45]. Therefore, the In_2_S_3_/Ti_3_C_2_/TiO_2_ ternary heterojunction can realize a dual photogenerated charge transfer channel due to the formation of the S-scheme and Schottky junction, which can effectively improve the separation and efficiency of the photogenerated charge. At the same time, the tight binding between nanosheets also shortens the transfer distance of photogenerated charge, thereby boosting the photocatalytic H_2_ production activity.

## 3. Materials and Methods

### 3.1. Preparation of Ti_3_C_2_ Nanosheets

A total of 1 g of Ti_3_AlC_2_ was slowly added into a 40 mL solution containing 2 g of LiF and 9 mol L^−1^ HCl, and the mixture was stirred at 35 °C for 24 h to etch the Al layers from Ti_3_AlC_2_. The resulting suspension was centrifuged and washed repeatedly with deionized water until the pH approached approximately 6. Next, the obtained black powder was redispersed in deionized water and treated by sonication for 3 h under a N_2_ atmosphere. Finally, centrifugation was performed at 3500× *g* rpm for 1 h to obtain few-layered Ti_3_C_2_ nanosheets.

### 3.2. Preparation of TiO_2_ Nanosheets

The 10 mL of tetrabutyl titanate was dropwise added into 5 mL of HF with continuous stirring at room temperature to form a homogeneous solution. Then, 30 mL of ethanol was added and followed by 20 min of stirring to achieve uniform dispersion. Thereafter, the dispersed solution was hydrothermally reacted at 180 °C for 16 h. After the reaction was completed, the sample was centrifuged and washed. Finally, the resulting powder was calcined at 500 °C for 2 h with a heating rate of 5 °C min^−1^ to obtain TiO_2_ nanosheets.

### 3.3. Preparation of In_2_S_3_/Ti_3_C_2_ Heterojunction

The In_2_S_3_/Ti_3_C_2_ heterojunction was prepared via the hydrothermal method. Typically, 0.01 g of Ti_3_C_2_ was added into 25 mL of deionized water and 25 mL of ethylene glycol to achieve homogeneous dispersion under a constant stirring condition. Then, 0.221 g of InCl_3_ 4H_2_O and 0.09 g of TAA were added to the above solution and stirred for 30 min. Subsequently, the above solution was subjected to a hydrothermal reaction at 140 °C for 8 h. The In_2_S_3_/Ti_3_C_2_ heterojunction was obtained after centrifugation, washing, and vacuum drying.

The mass ratios of Ti_3_C_2_ in the In_2_S_3_/Ti_3_C_2_ heterojunctions were adjusted to 3 wt%, 7 wt%, and 11 wt%, and the corresponding products were labelled as IM-3%, IM-7%, and IM-11%, respectively. Furthermore, pure In_2_S_3_ was also prepared via the same procedure without adding Ti_3_C_2_ nanosheets.

### 3.4. Preparation of In_2_S_3_/Ti_3_C_2_/TiO_2_ Ternary Heterojunction

The In_2_S_3_/Ti_3_C_2_/TiO_2_ ternary heterojunction was prepared using a self-assembly strategy. Typically, 0.1 g of TiO_2_ nanosheets and 0.1 g of the In_2_S_3_/Ti_3_C_2_ heterojunction (IM-7%) were added into 50 mL of deionized water, and ultrasonication was carried out for 1 h to make the dispersion uniform. After that, N_2_ was passed for 30 min and stirred for 12 h. Finally, the resulting precipitates were filtered, vacuum dried, and milled to obtain the In_2_S_3_/Ti_3_C_2_/TiO_2_ ternary heterojunction. Furthermore, the mass ratios of TiO_2_ in the In_2_S_3_/Ti_3_C_2_/TiO_2_ ternary heterojunctions were controlled at 30 wt%, 50 wt%, and 70 wt%, corresponding to IMT-30%, IMT-50%, and IMT-70%, respectively.

### 3.5. Photocatalytic H_2_ Production Test

Photocatalytic H_2_ production activity was evaluated in a Pyrex glass reaction vessel integrated with a gas-tight circulation system using a 300 W Xenon lamp as the light source (Beijing China Education Au-light Co., Ltd., Beijing, China). Typically, 20 mg of photocatalyst was dispersed into 100 mL of aqueous solution containing 20 vol% triethanolamine, followed by sonication for 10 min. Before light irradiation, the glass reactor was evacuated thoroughly to eliminate the air. The amount of H_2_ production was quantified using an on-line GC7920 gas chromatograph equipped with a thermal conductivity detector (Beijing China Education Au-light Co., Ltd., Beijing, China).

More details can be found in the Supplementary Materials.

## 4. Conclusions

In summary, the In_2_S_3_/Ti_3_C_2_/TiO_2_ ternary heterojunction with a hierarchical structure was successfully fabricated through a combination of the hydrothermal method and a self-assembly strategy. The resulting ternary heterojunction provides intimate interfacial contact and excellent structural stability. Under simulated sunlight irradiation, the optimized In_2_S_3_/Ti_3_C_2_/TiO_2_ ternary heterojunction exhibits an outstanding photocatalytic H_2_ production activity of 446.65 umol g^−1^ h^−1^, corresponding to about 22 and 20 times higher activity than that of the pure TiO_2_ and In_2_S_3_, respectively. The excellent photocatalytic H_2_ production activity is correlated with the dual photogenerated charge transfer channel, which is constructed by the S-scheme and Schottky junction in the ternary heterojunction and serves to facilitate the efficient separation and transfer of photogenerated electron–hole pairs. This study may open up a platform for improving the photocatalytic H_2_ production activity by integrating two different heterojunctions.

## Data Availability

The original contributions presented in this study are included in the article/Supplementary Materials. Further inquiries can be directed to the corresponding authors.

## Supplementary Materials

The following supporting information can be downloaded at: https://www.mdpi.com/article/10.3390/molecules31101751/s1. Figure S1. FESEM images of (a) TiO_2_ nanosheets and (b) Ti_3_C_2_ nanosheets. Figure S2. Photocatalytic H_2_ production curves of the In_2_S_3_/Ti_3_C_2_/TiO_2_ ternary heterojunction under visible light irradiation (λ > 420 nm). Figure S3. FESEM image of IMT-50% after the photocatalytic H_2_ production experiment. Figure S4. Schematic illustration of the charge-transfer pathway for a conventional type-II heterojunction. Table S1. The comparison of the photocatalytic H_2_ production rate of the In_2_S_3_/Ti_3_C_2_/TiO_2_ heterojunction with other reported photocatalysts. References [46,47,48,49,50,51,52,53] are cited in the Supplementary Materials.
