# Supplementary material for: Spatially Oriented S-Scheme and Schottky Junction in In2S3/Ti3C2/TiO2 Ternary Heterojunction for Efficient Photocatalytic H2 Production
Source: Molecules. 2026 May 20;31(10):1751. doi: 10.3390/molecules31101751 (PMC13209202; doi:10.3390/molecules31101751)
Supplement: Supplementary file 1 [file molecules-31-01751-s001.zip › molecules-4292423-supplementary.pdf]

molecules

*Article*

# Spatially Oriented S-Scheme and Schottky Junction in $In_2S_3/Ti_3C_2/TiO_2$ Ternary Heterojunction for Efficient Photocatalytic $H_2$ Production

**Wenyu Liu [1,2], Defa Liu [1], Bin Sun [1,*], Xingpeng Liu [1], Pengfei Gao [1], Xiao Lin [3] and Guowei Zhou [1,*]**

1 Key Laboratory of Fine Chemicals in Universities of Shandong, Jinan Engineering Laboratory for Multi-Scale Functional Materials, School of Chemistry and Chemical Engineering, Qilu University of Technology (Shandong Academy of Sciences), Jinan 250353, China; wenyuliu@qlu.edu.cn (W.L.); 10431241130@stu.qlu.edu.cn (D.L.); 10431231065@stu.qlu.edu.cn (X.L.); 10431251216@stu.qlu.edu.cn (P.G.)
2 Shandong Institute of Mechanical Design and Research, School of Mechanical Engineering, Qilu University of Technology (Shandong Academy of Sciences), Jinan 250353, China
3 College of Chemistry and Chemical Engineering, Jishou University, Jishou 416000, China; linxiao2017@whu.edu.cn
* Correspondence: binsun@qlu.edu.cn (B.S.); gwzhou@qlu.edu.cn (G.Z.)

# S1. Materials and Methods

## *S1.1. Materials*

$Ti_3AlC_2$ powder (purity > 99%, 400 mesh) was obtained from Jilin 11 Technology Co., Ltd., China. Indium chloride tetrahydrate ($InCl_3$ $4H_2O$) and lithium fluoride (LiF) were supplied by Shanghai Aladdin Chemistry Co., Ltd., China. Thioacetamide, tetrabutyl titanate, ethanol, hydrofluoric acid, ethylene glycol, and triethanolamine were acquired from Sinopharm Chemical Reagent Co., Ltd., China

## *S1.2. Characterization*

The crystal structures of as-synthesized samples were determined using a Bruker D8-Advance X-ray powder diffractometer (XRD) with a scan rate of 0.1° $s^{-1}$ and a $2\theta$ range of 5° to 80°. The morphology, microstructure, and elemental analysis of as-obtained samples were acquired by field emission scanning electron microscopy (FESEM, Hitachi Regulus8220) and transmission electron microscope (TEM, JOEL JEM-2100) coupled with an energy dispersive spectrometer (EDS). The surface chemical composition and valence state were analyzed via X-ray photoelectron spectroscopy (XPS, Thermo Scientific ESCA-Lab 250Xi) using 200 W monochromatic Al K$\alpha$ radiation. The UV-vis diffuse reflectance spectra were measured on a Shimadzu UV 2600 equipped with an integrating sphere. The photoluminescence (PL) spectra were measured by the F-4600 fluorescence spectrophotometer at 310 nm excitation wavelength. In-situ electron paramagnetic resonance (EPR) spectra were using a Bruker EMXplus to analyze the DMPO-•OH and DMPO-•$O_2^-$.

## *S1.3. Photoelectrochemical Test*

The transient photocurrent response, electrochemical impedance spectroscopy (EIS), linear sweep voltammetry (LSV), and Mott-Schottky test of as-synthesized samples were conducted using the CHI760E electrochemical workstation (Shanghai Chenhua, China). A standard three-electrode configuration was adopted with 0.5 mol $L^{-1}$ $Na_2SO_4$ aqueous solution as the electrolyte, where the as-obtained samples served as the working electrode, a Pt plate as the counter electrode, an Ag/AgCl as the reference electrode, and a 300 W Xenon lamp as light source, respectively. The working electrode was fabricated as follows: 10 mg of as-prepared photocatalyst powder, 0.5 mL of ethanol, and 80 μL of Nafion were mixed to form a suspension via sonication treatment. The suspension was then deposited onto indium-tin oxide glass (1 cm × 2 cm) by a drop-casting method, followed by drying the electrode in a vacuum oven at 60 °C for 5 h.

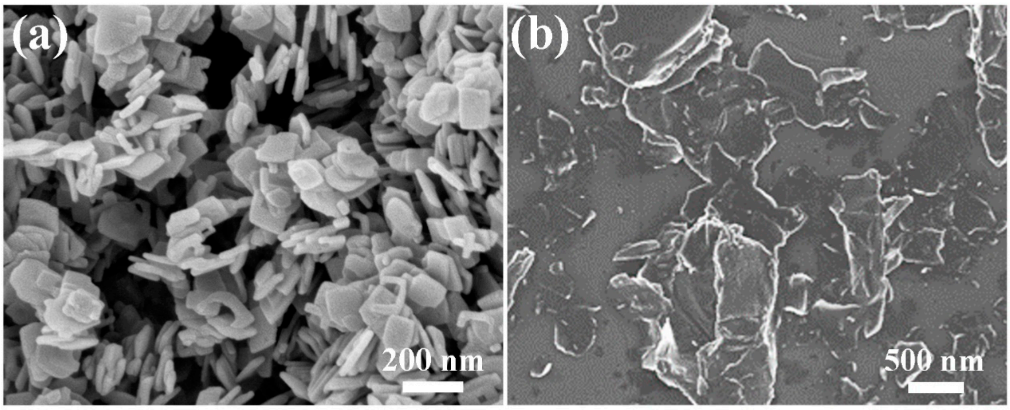


**Figure S1.** FESEM images of (**a**) $TiO_2$ nanosheets and (**b**) $Ti_3C_2$ nanosheets.

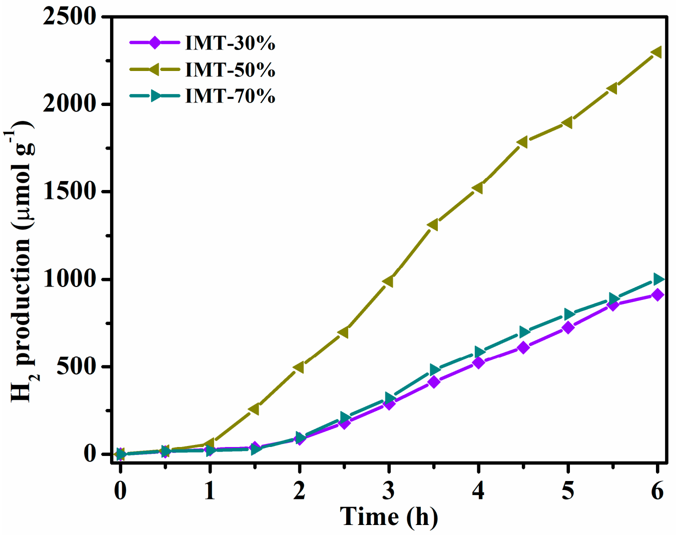


**Figure S2.** Photocatalytic $H_2$ production curves of $In_2S_3/Ti_3C_2/TiO_2$ ternary heterojunction under visible light irradiation ($\lambda$>420 nm).

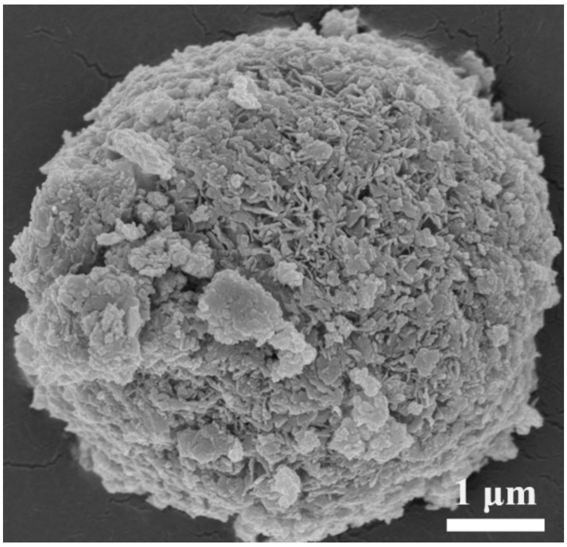


**Figure S3.** FESEM image of IMT-50% after photocatalytic $H_2$ production experiment.

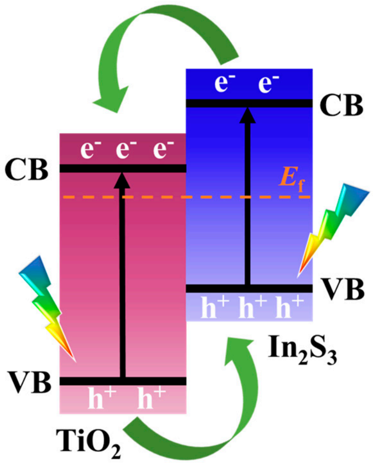


**Figure S4.** Schematic illustration of the charge-transfer pathway for a conventional type-II heterojunction.

https://doi.org/10.3390/xxxxx

**Table S1.** The comparison of photocatalytic $H_2$ production rate of $In_2S_3/Ti_3C_2/TiO_2$ heterojunction with other reported photocatalysts.

| Photocatalyst | Sacrificial agent | $H_2$ production rate ($\mu$mol $g^{-1}$ $h^{-1}$) | Ref. |
|---|---|---|---|
| $In_2S_3/WO_3$ | TEOA | 390 | [1] |
| $In_2S_3/C_3N_4$ | TEOA | 414.5 | [2] |
| $PdS/In_2S_3$ | $Na_2SO_3/Na_2S$ | 142.27 | [3] |
| Ni-$TiO_2$/g-$C_3N_4$ | TEOA | 134 | [4] |
| C/$TiO_2$ | Methanol | 314 | [5] |
| $TiO_2$/g-$C_3N_4$ | TEOA | 286 | [6] |
| $Ti_3C_2$ MXene@$TiO_2/CuInS_2$ | Methanol | 356.27 | [7] |
| $Ti_3C_2(RSO_3H)_x/TiO_2$ | Ethanol | 90.8 | [8] |
| $In_2S_3/Ti_3C_2/TiO_2$ | TEOA | 446.65 | This work |

## References


1. Lin, Y.; Chen, L.; Zhang, J.H.; Gui, Y.Y.; Liu, L.J. Hierarchical $In_2S_3$ microflowers decorated with $WO_3$ quantum dots: Sculpting S-scheme heterostructure for enhanced photocatalytic $H_2$ evolution and nitrobenzene hydrogenation. *J. Mater. Sci. Technol.* **2024**, *174*, 218–225.
2. Liu, Y.P.; Li, X.D.; Li, Z.G.; Chen, J.G.; Fang, B.Z. A dual reduction-type semiconductor heterojunction of $In_2S_3/C_3N_4$ for improved photocatalytic hydrogen production under visible light. *J. Alloy. Compd.* **2026**, *1052*, 186085.
3. Zhang, R.Y.; Jia, X.W.; Li, Y.R.; Yu, X.D.; Xing, Y. Oxidation co-catalyst modified $In_2S_3$ with efficient interfacial charge transfer for boosting photocatalytic $H_2$ evolution. *Int. J. Hydrogen Energy* **2022**, *47*, 25300–25308.
4. Yang, S.Y.; Wang, K.L.; Chen, Q.; Wu. Y. Enhanced photocatalytic hydrogen production of S-scheme $TiO_2$/g-$C_3N_4$ heterojunction loaded with single-atom Ni. *J. Mater. Sci. Technol.* **2024**, *175*, 104–114.
5. Zhao, Y.; Yang, N.; Zhou, T.; Zhan, W.J.; Zhao, J.H.; Chen, M.P.; He, T.W.; Zhang, J.; Zhang, Y.M.; Zhang, G.L.; Liu, Q.J. Mechanism and performance of photocatalytic $H_2$ evolution for carbon self-doped $TiO_2$ derived from MIL-125. *Int. J. Hydrogen Energy* **2024**, *65*, 151–157.
6. Sundaram, I.M.; Kalimuthu, S.; Sekar, K.; Rajendran, S. Hierarchical $TiO_2$ spheroids decorated g-$C_3N_4$ nanocomposite for solar driven hydrogen production and water depollution. *Int. J. Hydrogen Energy* **2022**, *47*, 3709–3721.
7. Yang, W.X.; Ma, G.Z.; Fu, Y.; Peng, K.; Yang, H.L.; Zhan, X.Q.; Yang, W.Y.; Wang, L.; Hou, H.L. Rationally designed $Ti_3C_2$ MXene@$TiO_2/CuInS_2$ Schottky/S-scheme integrated heterojunction for enhanced photocatalytic hydrogen evolution. *Chem. Eng. J.* **2022**, *429*, 132381.
8. Chen, T.T.; Wang, P.; Wang, X.F.; Chen, F. Surface terminal engineering of Ti sites in $Ti_3C_2$ MXene to weaken H adsorption for efficient photocatalytic $H_2$ evolution. *Int. J. Hydrogen Energy* **2026**, *202*, 152869.
